# Metformin increases antitumor activity of MEK inhibitors through GLI1 downregulation in LKB1 positive human NSCLC cancer cells

**DOI:** 10.18632/oncotarget.6559

**Published:** 2015-12-11

**Authors:** Carminia Maria Della Corte, Vincenza Ciaramella, Concetta Di Mauro, Maria Domenica Castellone, Federica Papaccio, Morena Fasano, Ferdinando Carlo Sasso, Erika Martinelli, Teresa Troiani, Ferdinando De Vita, Michele Orditura, Roberto Bianco, Fortunato Ciardiello, Floriana Morgillo

**Affiliations:** ^1^ Oncologia Medica, Dipartimento Medico-Chirurgico di Internistica Clinica e Sperimentale “F. Magrassi e A. Lanzara”, Seconda Università degli Studi di Napoli, Naples, Italy; ^2^ Oncologia Medica, Dipartimento di Medicina Clinica e Chirurgia, Università degli studi di Napoli “Federico II”, Naples, Italy; ^3^ Dipartimento di Medicina Molecolare e Biotecnologie Mediche, Istituto di Endocrinologia ed Oncologia Sperimentale “G. Salvatore” (IEOS), University of Naples “Federico II”, Naples, Italy; ^4^ Medicina Interna, Dipartimento Medico-Chirurgico di Internistica Clinica e Sperimentale “F. Magrassi e A. Lanzara”, Seconda Università degli Studi di Napoli, Naples, Italy

**Keywords:** metformin, MEK, selumetinib, pimasertib, NSCLC

## Abstract

**Purpose:**

Metformin, widely used as antidiabetic drug, showed antitumoral effects expecially in combination with chemotherapy. Our group recently has demonstrated that metformin and gefitinib are synergistic in *LKB1*-wild-type NSCLC cells. In these models, metformin as single agent induced an activation and phosphorylation of mitogen-activated-protein-kinase (MAPK) through an increased C-RAF/B-RAF heterodimerization.

**Experimental design:**

Since single agent metformin enhances proliferating signals through the RAS/RAF/MAPK pathway, and several MEK inhibitors (MEK-I) demonstrated clinical efficacy in combination with other agents in NSCLC, we tested the effects of metformin plus MEK-I (selumetinib or pimasertib) on proliferation, invasiveness, migration abilities *in vitro* and *in vivo* in LKB1 positive NSCLC models harboring *KRAS* wild type and mutated gene.

**Results:**

The combination of metformin with MEK-I showed a strong anti-proliferative and proapoptotic effect in Calu-3, H1299, H358 and H1975 human NSCLC cell lines, independently from the *KRAS* mutational status. The combination reduced the metastatic behaviour of NSCLC cells, via a downregulation of GLI1 trascritional activity, thus affecting the transition from an epithelial to a mesenchymal phenotype. Metformin and MEK-Is combinations also decreased the production and activity of MMP-2 and MMP-9 by reducing the NF-jB (p65) binding to MMP-2 and MMP-9 promoters.

**Conclusions:**

Metformin potentiates the antitumor activity of MEK-Is in human *LKB1*-wild-type NSCLC cell lines, independently from the *KRAS* mutational status, through GLI1 downregulation and by reducing the NF-jB (p65)-mediated transcription of MMP-2 and MMP-9.

## INTRODUCTION

Non-small cell lung cancer (NSCLC) is the major cause of cancer-related deaths worldwide [1].

Platinum-based combination regimens offer a significant although modest survival advantage to patients with stage IV NSCLCs [2]. Advances in the understanding of the molecular biology of cancer have enabled the discovery of several potential molecular targets with the development of novel targeted therapies and new combinatorial strategies.

Metformin (N0, N0-dimethylbiguanide) belongs to the biguanide class of oral hypoglycemic agents and is a widely used antidiabetic drug, now prescribed to almost 120 million people in the world for the treatment of type II diabetes [3]. Metformin also displays significant growth-inhibitory and proapoptotic effects in several cancer models, alone [4–7] or in combination with chemotherapeutic drugs [8, 9]. A recent study showed that metformin prevents tobacco-induced carcinogenesis in mice with 72% decrease in tumor burden [10]. The effects of metformin on cancer cell proliferation have been associated with AMPK activation, reduced mTOR signaling, and protein synthesis [11, 12]. Our group recently demonstrated the evidence of a significant synergism of metformin with gefitinib, a selective EGFR tyrosine kinase inhibitor (EGFR-TKI) on NSCLC cell lines [13]. For this purpose, a panel of human NSCLC cell lines with a defined spectrum of sensitivity to gefitinib was used [14].

The combination of metformin with gefitinib strongly reduced the anchorage-independent colony-forming ability and proliferation of NSCLC cell lines harboring wild type *LKB1* gene. Such effects were also shown in those NSCLC cell lines resistant to the EGFR-TKI, suggesting that metformin can revert resistance to gefitinib in some cancer cell lines. The combined treatment also demonstrated a strong proapoptotic effect and a pronounced decrease in the activation of key intracellular mediators of cell survival and proliferation signals such as MAPK and Akt. The combined treatment also affected the mTOR signaling as suggested by the sustained inhibition of the phosphorylation of S6 and of p70S6K [13]. Of interest, single-agent metformin treatment caused an unexpected increase in the levels of activated phosphorylated MAPK as a result of an increased B-RAF and C-RAF association [13] mediated by the inactivation of Rheb. Indeed, coimmunoprecipitation experiments revealed an increased B-RAF and C-RAF association, which could be responsible for the activation of MAPK after metformin treatment.

This is therapeutically relevant, since it has been shown that, while exerting antiproliferative and proapoptotic effects in combination with EGFR-inhibitors, single agent metformin treatment could enhance proliferating signals through the RAS/RAF/MAPK pathway, that could in turn induce cell proliferation in those cell lines with constitutively activating Ras mutations. This consideration opens new possibilities for combination of metformin with MEK inhibitors.

Currently a number of highly specific and highly potent MEK1/2 inhibitors (MEK-I) have been developed and evaluated in clinical studies. Most of these agents have shown moderate single agent activity in various tumors and in lung cancer in particular [14–17]. Among the factors contributing to the observed lack of clinical efficacy of MEK inhibitors, the activation of alternative pathways downstream of RAS and/or RAF, such as PI3K–AKT, could potentially compensate for the effects of MEK inhibition and eliminate the antitumour activity of MEK inhibitors in RAS–RAF-driven malignancies [18, 19].

Recently, Jänne and colleagues showed that the combination of the MEK inhibitor, selumetinib, and docetaxel have a synergistic effect in advanced *KRAS*-mutated NSCLC [20, 21].

The aim of this work was to examine the effects of metformin, in the presence of MEK inhibition, on a panel of NSCLC cell lines harboring *KRAS* wild type and mutated gene.

## RESULTS

### Synergistic effect of metformin and MEK inhibitor on NSCLC cell lines

**Figure 1:**
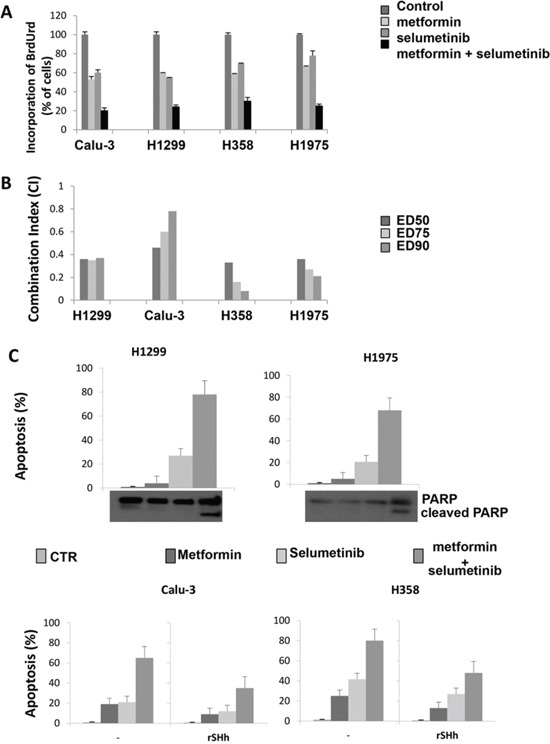
Effect of metformin alone and in combination with selumetinib on cell proliferation, on the induction of apoptosis and activation of GLI1 in CALU-3, H1299, H358 and H1975 cell lines **A.** Effect of metformin alone and in combination with selumetinib on cell proliferation in CALU-3, H1299, H358 and H1975 cell lines. Cells were treated with metformin, selumetinib and combination of both. Cell proliferation was measured by BrdUrd incorporation assay. BrdUrd was added for 1 hour, and cells were processed for immunofluorescence with anti-BrdUrd. Cell nuclei were counterstained with Hoechst. The average results ± SD of 3 independent experiments in which at least 500 cells were counted are shown. **B.** Combination index (CI) values from CALU-3, H1299, H358 and H1975 cell lines treated with metformin alone and in combination with selumetinib obtained with CompuSyn Program for different doses. ED50, ED75 ED90 represent the doses effecting 50, 75, and 90%, respectively of growth inhibition compared to control. **C.** Apoptosis was evaluated as described in Materials and Methods with Annexin V staining in CALU-3, H1299, H358 and H1975 cancer cells, which were treated, in the absence or presence of recombinant Sonic Hedgehog, with metformin, selumetinib or both. Columns mean of 3 identical wells of a single representative experiment. Western Blot analysis for PARP, (89)-cleaved-PARP fragment were performed on protein lysates from cell after the indicated treatment.

To evaluate the antiproliferative effects of metformin in combination with a MEK-inhibitor, we measured the inhibition of cell proliferation by using the BrdUrd incorporation of cells treated with single treatments with metformin or selumetinib, a selective MEK-inhibitor (MEK-I), and their combination (Figure 1A). To this end we used two *KRAS*-mutant NSCLC cell lines, H358 (*KRAS G12C*) and Calu-3 (*K-RAS G13D*), and two *KRAS*-wild type cell lines, H1299 (*NRAS Q61K* mutated) and H1975 (*EGFR T790M, L835R* mutated), as indicated in Table 1. In particular, NSCLC cell lines harbouring NRAS mutation correlate with major sensitivity to MEK-inhibitors, whereas cells with KRAS mutations show variable response [22].

**Table 1:** Mutational status and IC50 of metformin, selumetinib and pimasertib in our panel of NSCLC cell lines

Cell line	Mutation	Metformin IC50 (mmol/L)	Selumetinib IC50 (μM)	Pimasertib IC50 (μM)
H358	KRAS G12C	1.5	> 1	> 1
Calu-3	K-RAS G13D TP53 CDKN2A	1	0.5	0.5
H1299	NRAS Q61K	1.5	0.01	0.01
H1975	EGFR (T790M, L835R)	2.5	>5	>5

In addition, the cell line panel used in this work does not harbor any mutation in the LKB1 gene. We chose these cell lines harbouring *LKB1* wild-type gene since we previously demonstrated that metformin interferes and leads to activation of AMPK by LKB1 in the absence of *LKB1* mutation [13].

Different doses of metformin, alone and in combination with selumetinib, were studied; the cell lines, their mutations and IC50 values for each single drug are reported in Table 1. The IC50 values presented an average value of 2mmol/L for metformin and ranged from 0,01 to >10 μM for selumetinib and pimasertib.

Combined treatement of metformin and selumetinib exerted a strong antiproliferative effect as compared to single treatment alone (Figure 1A). To quantify the effect of the combined therapy, we used CompuSyn software to calculate the CI in all NSCLC cell lines. All cell lines had a CI index between 0.08 and 0.7 indicating synergism according to the method of Chou-Talalay [23] (Figure 1B). No cell line showed an antagonistic effect to the combination therapy. Of interest, also proliferation of those cell lines with relatively low sensitivity to selumetinib, H358 and H1975, resulted strongly decreased when combined treatment with metformin was performed. Similar results were obtained with another MEK-I inhibitor, pimasertib (data not shown).

We further asked whether the increased antiproliferative effect induced by the combined therapy of metformin and selumetinib would be the result of an increased apoptosis. Therefore, we analyzed the induction of apoptosis in Calu-3, H1299, H1975 and H358 human NSCLC cell lines after 72-hour treatment with metformin and selumetinib. As shown in Figure 1C, flow cytometric analysis revealed that combined treatment with the MEK-I and metformin significantly increased of several folds the percentage of apoptotic cells in all cell lines tested. For instance, Calu-3 cells presented respectively a 19% and 21% apoptotic rate in metformin- and selumetinib-treated cells, while the combination reached an apoptotic rate of 65% dying cells (*P* < 0.001; Figure 1C). These results were confirmed by Western blot analysis for PARP protein (Figure 1C): the combined treatment was able to induce the cleavage of the 113-kDa PARP to the 89-kDa fragments in all tested cell lines. These findings suggest that the treatment with metformin, concomitantly to the MEK inhibition, blocks proliferation and/or survival mechanisms in NSCLC cancer cells independently of the *KRAS* mutational status. To investigate if the apoptic effect depend on reduced GLI1 activation, we repeated the analysis in the presence of recombinant Sonic Hh and we found that reactivation of GLI1 in treated cells attenuates apoptosis induction (Figure 1C).

**Figure 2:**
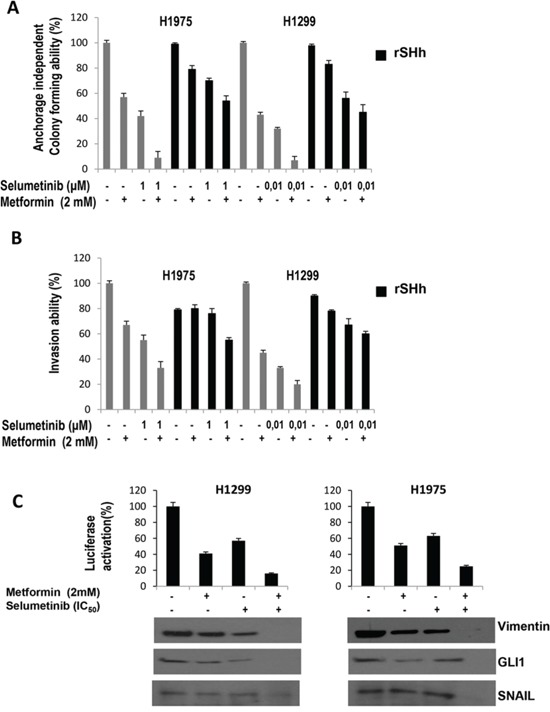
GLI1-mediated effects of metformin, selumetinib or both in NSCLC cell lines **A.** anchorage-independent colony formation in soft-agar; **B.** Invasion assay; in the absence or presence of recombinant Sonic Hedgehog; The results are the average ± SD of three independent experiments, each done in triplicate. **C.** GLI1-driven luciferase expression in H1299 cells before and after treatment with metformin, selumetinib or both. Western Blot analysis for EMT-related protein Vimentin and Snail and GLI1 were performed on protein lysates from cell after the indicated treatment.

One important characteristic of malignant cells is their ability to growth in semisolid medium, to invade and migrate. NSCLC cells display different behaviour: for instance, among the panel of NSCLC cells of this study, only H1299 and H1975 were able to migrate and invade. These cell lines were used to test the abilities of metformin and selumetinib to inhibit these characteristics. As shown in Figure 2A, and 2B, a significant dose-dependent inhibition of anchorage-independent colony forming ability and invasive aptitudes of H1299 and H1975 cell lines were observed following treatment of metformin combined with either selumetinib (Figure 2A and 2B) or pimasertib (data not shown). Similary data were obtained also for migratory abilities (data not shown). Considering the recent demonstrated role of GLI1 acivation as mediator of the epithelia-to-mesenchymal transition (EMT) and chemoresistance [24–28], and as previous studies demonstrated the effects of metformin on GLI1 activation in breast and pancreatic cancer models [29, 30], we analyzed the GLI1 trascription activity before and after treatment with metformin, MEK-I or both, in our model of NSCLC using a GLI1-responsive promoter within a luciferase reporter expression vector (Figure 2C). Analysis of luciferase acitivity following transfection of H1299 cells revealed a 6- to 7-fold decrease in GLI-responsive promoter activity by treatment with metformin and even further decrease by the combination of metformin and MEK-I, as compared to untreated H1299 cells (*P* < 0.001), suggesting that transcriptional activity of GLI1 is significantly inhibited by metformin alone and in combination. To investigate if the inhibition of GLI1 activity mediates the anti-metastatic effects of metformin, we analyzed the ability of the recombinant Sonic Hh to revert the metformin and MEK-I-mediated inhibition of invasion and migration abilities. Interestingly, activation of GLI1 reinduced, at least in part, cancer cells to invade and migrate in the presence of metformin or MEK-I alone and in combination (Figure 2A and 2B).

As mentioned above [29, 30], combined treatment decresed levels of GLI1 expression in NSCLC cell lines (Figure 2C). To investigate if EMT process, which has been recently associated to GLI1 activation in NSCLC cells with innate resistance to TKIs [28], is implicated in the sensitivity to the experimental combination we performed Western blot analysis for SNAIL and vimentin protein. These protein markers are known to induce EMT, which is strictly linked to a gain in migratory and invasive properties. Interestingly, the combination of metformin and selulmetinib was also able to increase SNAIL, that represses E-cadherin expression, and to decrease vimentin (Figure 2C).

### Effects of metformin and MEK inhibitor on intracellular pathways and MMP-9, MMP-2 and uPA expression and activity in NSCLC cells

To study the synergism obtained by the combination of metformin and selumetinib, western blot analyses were done on protein extracts from H1299 and H1975 NSCLC cells that were treated with 3mmol/L of metformin, the IC50 of selumetinib, or with combinations of both metformin and selumetinib. Treatment was conducted for 72 hours.

**Figure 3:**
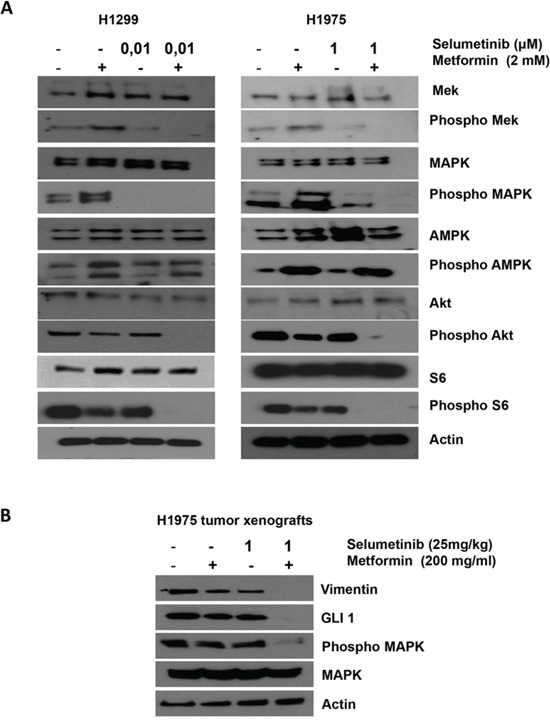
Effects of metformin, selumetinib or both on the downstream pathway in NSCLC **A.** Western blotting analysis of intracellular proteins MEK, MAPK, AMPK, Akt, S6, and their phosphorylated forms following treatment with the indicated concentration of metformin, selumetinib or both in H1299 and H1975 NSCLC cell line. β-Actin was included as a loading control. **B.** Western blotting analysis of Vimentin, GLI1, MAPK and its activated form on protein extracts from H1975 tumors harvested by mice treated with the indicated concentrations of metformin, selumetinib or their combination. β-Actin was included as a loading control.

We selected H1299 and H1975 cells to better investigate the synergism of experimental drugs in terms of invasive and migratory behavior. Figure 3A illustrates that metformin treatment, as single agent, although causing a decrease on the levels of activated phosphorylated AKT and S6 in both cell models, it mediates a paradoxical activation/phshorylation of MAPK already described in our previous work as a consequence of Rheb-dependent enhanced BRAF and C-RAF association [13]. Treatment with selumetinib as single agent decreased the level of activated MAPK without affecting the activation status of AKT and S6. Treatment with metformin in combination with selumetinib resulted in a concomitant decrease in the levels of protein phosphorylation (p-MAPK, p-AKT) (Figure 3A). The same results were obtained by Western blot analysis on tumoral sections derived from H1299 and H1975 xenografts (Figure 3B).

**Figure 4:**
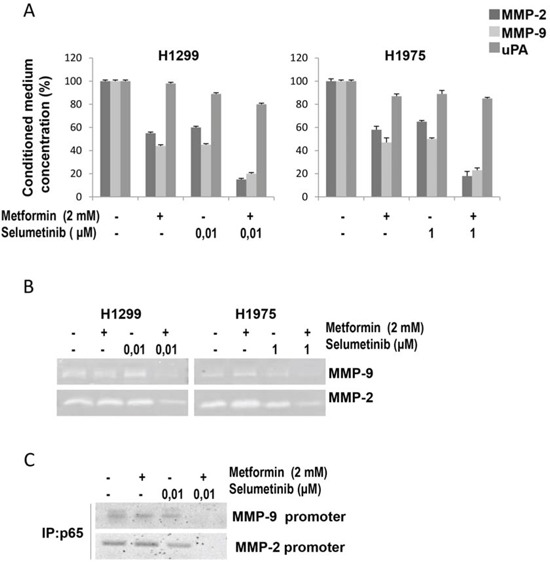
Effects of metformin and MEK inhibitor on intracellular pathways and MMP-9, MMP2 and uPA expression and activity in NSCLC cells **A.** Secretion of MMP-2, MMP-9 and uPA into the conditioned medium of H1299 and H1975 NSCLC cells, as measured in cell culture media by specific ELISAs. **B.** MMP-2 and MMP-9 activities determined by gelatin zymography in the conditioned media of H1299 and H1975 NSCLC cells. **C.** ChIP Assay evaluating the binding of NF-κB (p65) to the MMP9 and MMP2 promoters in H1299 cells.

In the context of EMT process, MMP-2, MMP-9 and uPA are thought to play a critical role in NSCLC cell migration and invasion by stimulating the degradation of extracellular matrix (ECM), and their increased expression is associated with disease progression. H1299 and H1975 cells displayed significant reduction in MMP-2, MMP-9 but not uPA protein production after combined treatment with metformin and selumetinib, as demonstrated by ELISA analysis in Figure 4A. The reduction in protein production corresponded to a reduced activity of MMP-2 and MMP-9, as determined by analysis of gelatin zymography (Figure 4B) while casein zymography did not evidenced any change in uPA activity. It is known that metformin significantly affects the nuclear localization of NF-κB (p65) [31] which is also a downstream target of MAPK. Therefore, we analyzed the binding capability of NF-κB (p65) on the promoters of MMP-2 and MMP-9 genes in the presence of metformin, selumetinib or both. ChIP analysis showed that when metformin is combined with the MEK-I, NF-κB (p65) binding to MMP-2 and MMP-9 promoter regions is significantly decreased in H1299 (Figure 4C) and H1975 (data not shown) cell lines.

### *In vivo* effects of the combined treatment with metformin and selumetinib

**Figure 5:**
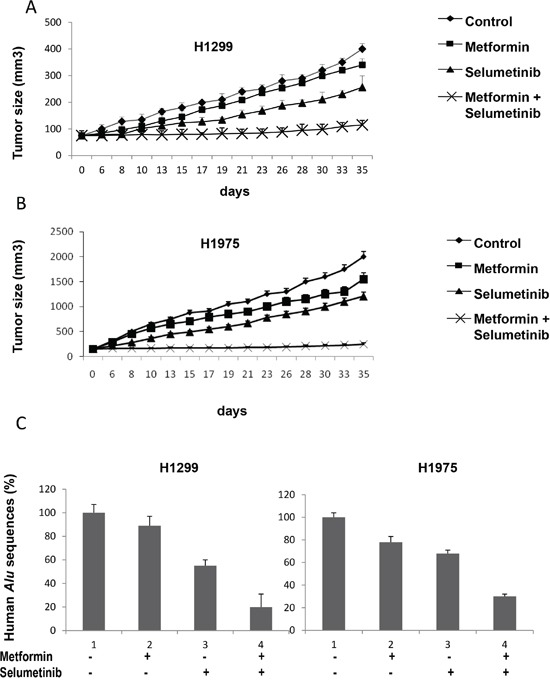
*In vivo* effects of the combined treatment with metformin and selumetinib **A, B.** Athymic nude mice were injected subcutaneously into the dorsal flank with 10^7^ NSCLC cancer cells. When the average tumor size was 75 mm^3^ in H1299 xenografts (A) and 150 mm^3^ in H1975 xenografts (B), mice were treated as indicated in Materials and Methods. Xenografted mice received only vehicle (control group), metformin (200 mg/mL metformin diluted in drinking water and present throughout the treatment period), selumetinib (25 mg/kg p.o.), or their combination. Data represent the average ± SD. Student *t* test was used to compare tumor sizes among different treatment groups at day 35 following the start of treatment. **C.** Percentage of human *Alu* sequences in the lungs of mice after tail vein injection with H1299 and H1975 cells and the indicated treatments.

We finally investigated the *in vivo* antitumor activity of metformin in nude mice bearing H1299 or H1975 cells that were grown subcutaneously as tumor xenografts. Treatment with metformin or selumetinib, as single agents, caused a slight decrease in tumor size as compared with control untreated mice. Treatment with the combination of metformin and selumetinib induced a significant reduction in tumor growth (Figure 5A and 5B). In this respect, at day 35 from the starting of treatment, the mean tumor volumes in the combination treatment group ranged between 60% and 20% in mice bearing H1299 tumor xenografts and between 50% and 12,5% in H1975 xenografts, as compared with their control untreated mice. During our experiments, no obvious side effects were observed in mice treated with metformin.

As our *in vitro* studies revealed expression changes of mesenchymal proteins, SNAIL and vimentin (Figure 2C), and of MMP-2 and MMP-9 on cancer cells treated with the combination of metformin and selumetinib (Figure 4), we also investigated whether the combination therapy blocks tumour metastatic behavior *in vivo*. Therefore, we performed an artificial metastasis assay by injecting H1299 or H1975 cells into the tail vein of Balb/c nude mice (six mice per group), and treating them with metformin or selumetinib alone, or in combination. To measure lung micrometastasis formation, we quantified the portion of human DNA in mouse lungs using real-time PCR for human Alu sequences, as previously described [32]. Untreated mice showed a detectable amount of human DNA in their lungs. No other site of distant metastases was observed in other organs (liver, spleen, brain, and bone). As expected, the combination of metformin with selumetinib was much more effective than single drugs in reducing levels of human DNA in mouse lungs (Figure 5C).

## DISCUSSION

As we prevoiusly demonstrated, the combination of metformin and gefitinib, a selective EGFR-TKI, showed a significant potentiation of the antiproliferative and proapoptotic activity both *in vitro* and *in vivo* in NSCLC cell lines [13]. However, in the same study, an increase in the level of activated phosphorylated MAPK after metformin treatment was observed.

Indeed, the metformin-induced activation of AMPK enhanced the C-RAF/B-RAF dimerization through a downregulation of Rheb, thus potentiating the signaling from RAS to MAPK. This could be therapeutically relevant, as we have shown that, while exerting antiproliferative and proapoptotic effects, single-agent metformin treatment could enhance pro-proliferating signals through the RAS/RAF/MAPK pathway, that could in turn induce cell proliferation in those cell lines with constitutively activating RAS mutations. The activation of MAPK by metformin treatment is the new scenario that we have to consider when testing metformin as anticancer agent. This consideration leads us to analyze new possibilities of treatment combination, in particular, metformin and MEK inhibitors.

We tested the antiproliferative and pro-apoptotic effects of the metformin and MEK-I (pimasertib or selumetinib) combination in a panel of NSCLC cell lines harbouring wild type *LKB1*, which has been defined as a predictor of response to metformin activity [13] and, in this study, we demonstrated not only that the synergism is evident in all the cell lines tested irrespective of the mutational status of *KRAS* gene, but the addition of metformin is able to overcome the innate resistance to MEK-Is, also in those cells with *KRAS* mutation, such as H358 cell line. The mechanism of anti-tumoral effects of experimental combination include the inhibition of GLI1 transcriptional activity and synthesis, as showed in Figure 2C and as demonstrated by the attenuation of anti-apoptic effect with GLI1 reactivation (Figure 1C). Based on the results from previous studies [13] we are currently running a phase I-II study of combination of metformin and erlotinib in second-third line therapy of NSCLC patients [33] unselected for *EGFR* activating mutations. With the introduction of MEK-Is in the management of NSCLC patients and if this study will provide positive results, the combination of metformin and MEK-I will represents a new challenge of therapy in this setting of patients.

Furthermore, the efficacy of the combination had also a resonance in terms of invasion and migration inhibition: indeed, metformin alone exerted a potent anti-metastatic effect that was strongly enhanced by the addition of the MEK-inhibitor. The invasive and migratory abilities of cancer cells are particularly pronounced in poorly differentiated cancers or when drug-resistance occurs, and may represent an aspect of the EMT [34–36]. In our model of NSCLC cells, the experimental combination strongly reverted the expression of vimentin and SNAIL, which represent classical markers of EMT and correlate to a more aggressive behavior. Recently, we have reported a crucial role od Hedgehog pathway in mediating drug-resistance in a model of NSCLC harboring an activating mutation of EGFR gene and with acquired resistance to the first/second generation EGFR-TKIs [28]. In this model the aberrant activation of Hedgehog signaling, through an amplification of its main receptor SMO, lead to an over-activation of the main transcription factor GLI1, mediating the shift through a mesenchymal phenotype. For this reason we investigated if the anti-metastatic activity of metformin and its combination with MEK-Is was mediated by a decrease in the Hedgehog pathway activation [34, 35]. Of interest, metformin and MEK-I, as single agents, affected the GLI1 activation status, as evidenced by luciferase assay, and their combination resulted in an even stronger decrease in its transcriptional activity (Figure 2C). Additionaly, treatment with the recombinant Sonic Hedgehog partially reverted the drugs efficacy indicating that the combination's anti-metastatic activity is, at least in part, mediated by a secondary inhibition of GLI1.

In addition, as MMPs and uPA play a key role in degrading the extracellular matrix allowing metastatic cells to have access to the vasculature, and as their expression can be modulated by various intracellular upstream signaling cascades, particularly the MAPK pathway, we asked if metformin alone and in combination with MEK-Is further affected the secretion of such enzymes [31]. Consistent with this hypothesis, in this study, the secretion and expression levels of MMP-9 and MMP-2 of NSCLC cells, but not uPA, were found decreased after being treated with single agents and to a higher extent by the combination. Previous studies demonstrated the ability of metformin to decrease the nuclear translocation of NF-κB, which is a transcription factor usually activated in malignant tumor progression and altering gene expression patterns, leading to cancer metastasis [31, 37]. The synergistic combination of metformin and MEK-Is strongly inhibited the binding of NF-κB to the MMP9 and MMP2 promoters, thereby suppressing their expression and the metastatic potential of cancer cells. Consistently, our data suggest that the combined treatment of metformin and MEK-Is interferes with the EMT process and distant metastatis spread. Indeed, combined treatments not only significantly modulated EMT markers but also reduced the formation of lung micrometastasis in nude mice.

## MATERIALS AND METHODS

### Cell lines, drugs, and chemicals

The human NSCLC H1299, H358, H1975 and Calu-3 cell lines were provided by American Type Culture Collection (ATCC, Manassas, VA, USA) and maintained in RPMI 1640 (Sigma-Aldrich) medium supplemented with 10% fetal bovine serum (FBS; Life Technologies, Gaithersburg, MD) in a humidified atmosphere with 5% CO2. The identity of all cell lines was confirmed by STR profiling (Promega) on an ad hoc basis prior to performing experiments, and repeated after the majority of the experiments were performed.

Metformin was purchased from Sigma-Aldrich; Selumetinib (AZD6244) and Pimasertib (AS-703026) from Selleck Chemicals (Selleckchem, Houston, TX, USA). They were dissolved in sterile dimethylsulfoxide (DMSO) and a 10 mM stock solution was prepared and stored in aliquots at −20°C. Working concentrations were diluted in culture medium just before each experiment.

Recombinant Sonic Hedgehog was provided by Sigma-Aldrich.

Primary antibodies for western blot analysis against p-EGFR (Tyr1068), EGFR, p-MEK1/2 (Ser217/221), MEK1/2, p-MAPK44/42 (Thr202/Tyr204), MAPK44/42, p-AKT (Ser473), AKT, AMPK, p-AMPK (thr172), S6, p-S6 (Ser235-236), Vimentin, Snail, GLI1 and β-Actin were obtained from Cell Signaling Technology. The following secondary antibodies from Bio-Rad were used: goat anti-rabbit IgG and rabbit anti-mouse IgG.

### Cell proliferation assays

Cancer cells were seeded in 96-well plates and were treated with different doses of metformin, selumetinib or pimasertib or both for 72 hours.

DNA synthesis was measured by 5-bromo-20-deoxyuridine (BrdUrd) labeling and detection kit (Roche Diagnostics). Briefly, cells were seeded onto glass coverslips and treated for 72 hours. Then, cells were incubated for 1 hour with BrdUrd (10 mmol/L) and fixed. Coverslips were incubated with anti-BrdUrd and secondary fluorescein-conjugated antibody. The fluorescent signal was visualized with an epifluorescent microscope (Axiovert 2, Zeiss) interfaced with the image analyzer software KS300. Cell nuclei were counterstained with Hoechst. IC50 were determined by interpolation from the dose-response curves. Results represent the median of three separate experiments, each performed in quadruplicate. Synergism was calculated with ComboSyn software, ComboSyn Inc., Paramus, NK. 07652 USA.

### Protein expression analysis

Following treatment, cancer cells were lysed with Tween-20 lysis buffer (50 mmol/L HEPES, pH 7.4, 150 mmol/L NaCl, 0.1% Tween-20, 10% glycerol, 2.5 mmol/L EGTA, 1 mmol/L EDTA, 1 mmol/L DTT, 1 mmol/L phenylmethylsulfonylfluoride, and 10 μg/mL of leupeptin and aprotinin) and protein lysates containing comparable amounts of proteins, estimated by a modified Bradford assay (Bio-Rad), were subjected to western blot. Immunocomplexes were detected with the enhanced chemiluminescence kit ECL plus, by Thermo Fisher Scientific (Rockford, IL). Tumor samples harvested from mice were cut into 25 mm^3^ pieces and stored in RNA later until protein extraction for western blot analysis. Protein lysates were obtained by homogenization in RIPA lyses buffer (0.1% sodium dodecylsulfate (SDS), 0,5% deoxycholate, 1%Nonidet, 100mmol/L NaCl, 10 mmol/L Tris–HCl (pH 7.4), 0.5 mmol/L dithiotritol, and 0.5% phenylmethyl sulfonyl fluoride, protease inhibitor cocktail (Hoffmann-La Roche)) and clarification by centrifugation at 14,000 rpm for 10 minutes a 4°C.

### Luciferase assay

Luciferase assay was performed by using the Dual-Luciferase Assay system (Promega) following the manufacturer's protocol. The GLI-Luc reporter plasmid was kindly provided by Maria Domenica Castellone [38]. A total of 5×10^5^ cells were plated 24h before transfection in a 24 multiwell plate. The GLI-Luc reporter was transfected together with pRL-TK, encoding the Renilla luciferase (Promega), in triplicate, using FuGENE (Roche, Cat. No. 1815091) with the luciferase reporter. Luciferase activity was determined 48 h after transfection by using an Autolumat LB 953 (EG&G, Berthold, Bad Wildbad, Germany). Activity was reported as fold change with respect to control cells and cells transfected with the empty vector; results were the average of three independent experiments.

### Growth in soft agar

Cells (10^4^ cells/well) were suspended in 0.5 mL 0.3% Noble agar (Sigma-Aldrich) dissolved in complete culture medium. This suspension was layered over 0.5 mL 0.8% agar-medium base layer in 12 multiwell plate and daily treated with different concentrations of each drug alone or in combination. When tumor cell colonies were at least 80 μm, they were counted by using a dissection microscope. Assays were performed in triplicate.

### Invasion assay

The *in vitro* invasive ability of cells was measured by using transwell chambers (Corning Life Sciences, MA, USA) according to the manufacturer's protocol. Briefly, cells were seeded onto the membrane of the upper chamber of the transwell at a concentration of 5×10^4^/ml in 500 μl of RPMI medium and were treated with the indicated concentrations of each drug alone and in combination for 24 hours. The medium in the upper chamber was serum-free. The medium at the lower chamber contained 10% FBS as a source of chemo-attractants. Cells that passed through the Matrigel coated membrane were stained with Cell Stain Solution containing crystal violet (Chemicon, Millipore, CA, USA) and photographed after 24 hours. Absorbance was measured at 562 nm by an ELISA reader after dissolving of stained cells in 10% acetic acid. Assays were performed in triplicate.

### Migration assay

Cell migration was assessed using a commercially available chemotaxis assay. Briefly, cells were incubated in RPMI serum-free medium for 24 hours were left untreated or treated with the indicated treatments, following which they were detached from flasks, suspended in quenching medium (serum-free medium containing 5% bovine serum albumin) and EDTA, and seeded into Boyden migration chamber inserts placed in a 24-well plate (Cell Biolabs, CA, USA), containing a microporous membrane with an 8-μm pore size. Inserts were placed over wells containing serum-free media plus chemo-attractant (10% FBS). After a 48-h treatment period, cells/media were discarded from the topside of the migration chamber insert and the chamber was placed in the wells of a new 24-well plate containing cell detachment solution. Following incubation for 30 min at 37°C, the insert was discarded, and a solution of lysis buffer and CyQuant GR dye was added to each well (Invitrogen, OR, USA). CyQuant is a green fluorescent dye that exhibits strong enhancement of fluorescence when bound to cellular nucleic acids released by the lysis buffer, enabling assessment of the relative number of migrated cells. Fluorescence was determined with a fluorimeter at 480/520 nm. Assays were performed in triplicate.

### Assessment of apoptosis

Apoptosis was detected by flow cytometry via the examination of altered plasma membrane phospholipid packing by lipophilic dye Annexin V as described elsewhere [13]. Briefly, treated cells were harvested by trypsin, washed twice with PBS, and were then resuspended in binding buffer at a concentration of 1 × 10^6^ cells/mL according to the manufacturer's instruction. Thereafter, 5 μL of Annexin V-FITC and 5 μL of propidium iodide were added into 100 μL of cell suspension and incubated for 30 min at room temperature in the dark. After adding 400 μL of binding buffer, labeled cells were counted by flow cytometry within 30 min. All early apoptotic cells (Annexin V–positive, propidium iodide–negative), necrotic/late apoptotic cells (double positive), as well as living cells (double negative) were detected by FACSCalibur flow cytometer and subsequently analyzed by Cell Quest software (Becton Dickinson). Argon laser excitation wavelength was 488 nm, whereas emission data were acquired at wavelength 530 nm (FL-1 channel) for FITC and 670 nm (FL-3 c3 channel) for propidium iodide.

### Elisa

The levels of MMP-9, 2 and uPA (ng/ml) into cell culture media were determined using a ‘sandwich’ ELISA kit (R&D Systems Inc, Minneapolis, MN, USA) according to the manufacturer's guidelines. The minimum detectable levels were less than 1.2 ng/ml.

### Zymography assay

Analysis of MMP-2, MMP-9 and uPA activities was assayed by gelatin (for MMP-2 and MMP-9) or casein plasminogen (for uPA) zymography, as described previously [31, 39]. Briefly, conditioned media from cells cultured in the absence of serum for 48 hours, with the indicated treatments, were collected. Samples were mixed with loading buffer and electrophoresed on 8 % SDS-polyacrylamide gel containing 0.1 % gelatin or casein. Electrophoresis was performed at 100 V for 3 h. Then the gels were washed twice for 10 min at room temperature in zymography washing buffer (2.5% Triton X-100 in double-distilled H2O) to remove SDS. Gels were incubated in substrate buffer (40 mmol/L of Tris-HCl, 10 mmol/L CaCl2, 0.02% NaN3 and 1% Triton X-100, pH 8.0) at 37°C for 18 h, stained with Coomassie blue R-250 (0.125 % Coomassie blue R-250, 0.1 % amino black, 50 % methanol, and 10 % acetic acid) for one hour and destained with destaining solution (20 % methanol, 10 % acetic acid, and 70 % double-distilled H2O).

### ChIP assay

Chromatin immunoprecipitation (ChIP) assay was performed as described previously [40, 41]. The major steps in the ChIP assay include the crosslinking of target protein to the chromatin DNA with formaldehyde, the breaking of the chromatin DNA into fragments (400–1200 bp), the immunoprecipitation (IP) of the protein-DNA complex with an antibody that recognizes the target protein. The DNA in IP product was amplified in PCR with the ChIP assay primers that are specific to the NF-kB binding site at −316/−15. The sequences of the primers specific to the promoter of uPA gene are 5′-AGCATGACAGCCTCCAGCCAAGTA-3′(forward), and 5′-ACGTGACCAGAACATAAACAGAGA-3′ (reverse), and the promoter of MMP-9 gene are 5′-GAGGCTGCTACTGTCCCCT-3′ (forward), and 5′-GCTAGGCAAGGCTGGGGA-3′ (reverse). PCR products were analyzed on 2 % agarose gels and images were analyzed with Storm 860 Molecular Imager scanner.

### Experimental metastasis assay

One day before inoculation with H1299 or H1975 cells, mice (six mice per group) started treated with metformin (200 mg/mL metformin diluted in drinking water and present throughout the treatment period), selumetinib (25 mg/kg p.o.), or their combinations. Mice were inoculated with 20 × 10^5^ cells via tail vein injection, and treatment with metformin, selumetinib or their combination was continued for 7 consecutive days. All mice were killed on day 21 [42]. Human DNA in mouse lungs was measured by quantifying Alu sequences through PCR, as previously described [32]

### Tumor xenografts in nude mice

Four- to 6-week old female balb/c athymic (nuþ/nuþ) mice were purchased from Charles River Laboratories. The research protocol was approved and mice were maintained in accordance with the Institutional Guidelines of the Second University of Naples Animal Care and Use Committee. Mice were acclimatized for 1 week before being injected with cancer cells and injected subcutaneously with 10^7^ H1299 or H1975 cells that had been diluted in 200 μL of Matrigel (Corning Life Sciences, MA, USA) 1:1 in culture medium. When established tumors reached the volume of approximately 75 mm^3^ for H1299 xenografts and 150 mm^3^ for H1975 xenografts, mice were randomized in different groups (8 mice/group) of treatments: only vehicle (control group), metformin 200 mg/mL metformin diluted in drinking water and present throughout the treatment period), selumetinib (25 mg/kg p.o.), or their combination. Body weight and tumor volume were monitored on alternate days. Tumor volume was measured using the formula π/6 larger diameter × (smaller diameter)^2^.

### Statistical analysis

The Student *t* test was used to evaluate the statistical significance of the results. All *P* values represent 2-sided tests of statistical significance.
